# Hypertension and Cardiometabolic Risk Factors

**DOI:** 10.1155/2013/634798

**Published:** 2013-06-23

**Authors:** Mario Fritsch Neves, Agostino Virdis, Antonio Felipe Sanjuliani, Eduardo Vera Tibiriçá

**Affiliations:** ^1^Department of Clinical Medicine, State University of Rio de Janeiro, 20551030 Rio de Janeiro, RJ, Brazil; ^2^Department of Clinical and Experimental Medicine, University of Pisa, 56100, Italy; ^3^Department of Internal Medicine, State University of Rio de Janeiro, 20551030 Rio de Janeiro, RJ, Brazil; ^4^Laboratory of Cardiovascular Investigation, Oswaldo Cruz Institute, 21045900 Rio de Janeiro, RJ, Brazil

Despite the availability of nonpharmacological approaches and pharmacological therapies for hypertension, blood pressure control rates are disappointing all over the world [1]. Among several considerations, hypertension is usually associated with metabolic disorders, especially obesity, diabetes, and dyslipidemia, which may contribute to a greater difficulty of lowering blood pressure. In fact, the significant association between systemic hypertension and other cardiometabolic risk factors is well recognized, such as an elevated body mass index, waist circumference, fasting glucose, triglycerides, and a reduced HDL cholesterol, even not reaching criteria for diagnosis of metabolic syndrome. Individually, each one of these metabolic disorders is associated with adverse cardiovascular outcomes.

 This special issue presents some papers related to these cardiometabolic risk factors and their involvement mainly in prognosis and treatment of hypertensive patients. Firstly, a very low diastolic blood pressure, with a threshold value of 70 mmHg, was associated with increased all-cause mortality. It has been discussed that blood pressure level is the most important factor in cardiovascular risk evaluation even in hypertensive subjects with important metabolic disorders. The linear relationship between blood pressure and cardiovascular mortality starting from 115/75 mmHg is well known [2]. Nevertheless, cardiovascular events have also been observed after insistent lowering of blood pressure, at least in patients with coronary artery disease [3]. 

 Sleep disorders have been increasingly studied in recent years. Obstructive sleep apnea is related to cardiovascular disease and metabolic dysfunction, but sleep duration is a parameter not commonly considered in this association. Indeed, short sleep duration has been previously associated with an increased likelihood for hypertension [4]. In a paper of this collection, interactions of deficient sleep with race/ethnicity were noted even after adjusting to metabolic factors such as body mass index and diabetes. This was an interesting finding considering that insulin resistance exacerbated by renin angiotensin system (RAS) activation might be a mechanism involved in the link between sleep disorders and metabolic syndrome. The causal relationship of RAS and oxidative stress with vascular inflammation was documented in this special issue in a study with an experimental model of metabolic syndrome. In this protocol, a RAS inhibitor and an antioxidant agent were able to attenuate metabolic factors and systolic blood pressure in spontaneously hypertensive rats submitted to fructose administration. 

The interaction between hypertension and diabetes seems to be the principal concern to the cardiovascular system. The mechanisms that may increase cardiovascular risk in diabetic hypertensive patients are not completely understood. Oxidative stress and low-grade inflammation resulting in endothelial dysfunction as the first process of atherosclerosis were reviewed in this special issue. These factors may be also relevant in patients with longer duration of type 1 diabetes. In another paper, a cross-sectional study carried out in 20 Brazilian cities demonstrated that less than one quarter of treated type 1 diabetic patients achieved the target values for systolic and diastolic blood pressure. These data indicate that a more aggressive approach of this population is extremely necessary also in developing countries.

Nutritional factors involved in the treatment of hypertensive subjects are also discussed in this special issue. Many dietary components, especially sodium, potassium, calcium, and magnesium, are reviewed based on the available evidence. Restriction of daily sodium intake is recommended in several guidelines about hypertension. However, low salt intake has resulted in cardiovascular mortality in some particular groups of patients including diabetic subjects [5]. Potassium, calcium, and magnesium supplementations are not indicated as a usual practice for hypertensive subjects, but natural source of these micronutrients is the mainstay of the Dietary Approaches to Stop Hypertension (DASH) plan. Also, as a nonpharmacological approach, two original papers in this collection demonstrated the beneficial effects of dark chocolate consumption on endothelial function in specific populations: stage 1 hypertensive subjects with excess body weight and younger hypertensive individuals with low cardiovascular risk who already present endothelial dysfunction. It has been reported that these favorable outcomes are associated with reasonably high amount of polyphenol in dark chocolate.

 Lastly, in a study that enrolled patients with uncontrolled hypertension and criteria for metabolic syndrome, moxonidine therapy resulted in blood pressure reduction and improvement of blood pressure control rates, especially in younger individuals. The pharmacological treatment of hypertension in the context of metabolic syndrome presents many challenges in the daily clinical practice due to several mechanisms involved in this medical condition including the sympathetic nervous system (SNS) hyperactivation. Thus, moxonidine, a selective agonist at the imidazoline receptor subtype 1 which determines a decrease in SNS activity, may be beneficial in this circumstance. 

 Undoubtedly, hypertension is very commonly associated with other metabolic chronic conditions. In fact, more than 80% of hypertensive patients present one or more concomitant metabolic risk factors. This clinical condition has a growing global prevalence, clearly related to the modern lifestyles characterized by lack of physical activity resulting in overweight or obesity. Hopefully, we believe that this special issue can help to better understand the interaction between raised blood pressure levels and cardiometabolic factors, pointing out the involvement of insulin resistance and RAS/SNS activation as linking factors and also discussing some therapeutic options for this population. 

*Mario Fritsch Neves* *Mario Fritsch Neves* *Agostino Virdis* *Agostino Virdis* *Antonio Felipe Sanjuliani* *Antonio Felipe Sanjuliani* *Eduardo Vera Tibiriçá* *Eduardo Vera Tibiriçá*
